# Seroprevalence of Hepatitis B and Hepatitis C virus infection among HIV infected patients in Mumbai

**DOI:** 10.4103/2589-0557.75025

**Published:** 2010

**Authors:** Sandhya Sawant, Sachee Agrawal, Jayanthi Shastri

**Affiliations:** Department of Microbiology, B.Y.L. Nair Charitable Hospital, Mumbai, India

Sir,

Human Immunodeficiency Virus (HIV) infection appears to influence the natural history of infections with certain hepatitis viruses. Interactions between HIV and concurrent infections with hepatitis viruses may alter the natural history and treatment response of both diseases.[1] Hepatitis B Virus (HBV) and Hepatitis C Virus (HCV) have become major risk factors for mortality in patients receiving antiretroviral therapy(ART).[2] There is high degree of epidemiological similarity between HBV and HIV with regard to high risk groups, routes of transmission and the presence of virus in body fluids. Coinfection of HIV with HBV/HCV is known to result in higher viral load of hepatitis virus and greater liver damage.[1]

A study was carried out in the Department of Microbiology, Nair Hospital, from Januaryto April 2009, where clinically suspected patients were tested for HIV antibodies after pre-test counseling and informed consent, as per the guidelines by the National AIDS Control Organization (NACO), India. All HIV seropositive patients were tested for Hepatitis B surface Ag (HBsAg) and Anti HCV antibody by enzyme-linked immunosorbent assay(ELISA). Of the 540 HIV seropositive patients, 90(16.7%) were positive for HBsAg and 7(1.3%) were positive for HCV antibodies. Concomitant infection of HIV, HBV and HCV was found in 2(0.4%) patients. Heterosexual high risk behavior was observed in 435(80.6%) patients and history of blood transfusion in 15(2.8%) patients. Two patients(0.4%) who were intravenous drug users were positive for HIV, HBV and HCV. All 97 HIV seropositive coinfected patients were in the age group of 25–35 years. None of these patients gave history of perinatal transmission.

HIV shares common route of infection with HBV and HCV. HIV and HBV are known to be transmitted sexually. Sexual transmission of HCV appears to be certainly less efficient than is the case for HIV-1. However, sexual transmission of HCV has been documented. It is therefore not surprising to find that some patients with HIV are coinfected with HBV and/or HCV. HIV appears to have marked influence on the natural history of HBV infection. There is an increase in persistence of HBV, increase in HBV viral load and increase in the incidence of HBV reactivation and reinfection.[3]

The effect of HIV on HCV infection has not been investigated. Patients with HIV died long before their liver disease became problematic. With successful therapy of HIV, it is becoming clear that HCV may lead to early onset of advanced liver disease.[4] HIV infected individuals have a high probability of getting coinfected with HBV/HCV. HIV disease progression and enhanced immunosupression has a direct bearing on the natural history and pathogenesis of these infections. Sexual transmission of both HBV and HCV also appears to be significant and is of epidemiological importance in the light of heterosexual transmission of HIV in India.

Although screening for HBV and HCV infection in HIV infected patients is recommended in many HIV treatment guidelines, this screening has not been performed regularly in resource limited settings. Therefore, it may be necessary to integrate HIV and HBV/HCV care into the National AIDS Program and commence interventions and treatment guidelines for patients with HIV and HBV/HCV coinfection.
